# Combination therapy with proteasome inhibitors and TLR agonists enhances tumour cell death and IL-1β production

**DOI:** 10.1038/s41419-017-0194-1

**Published:** 2018-02-07

**Authors:** Anthony C Tang, Seyed M Rahavi, Shan-Yu Fung, Henry Y Lu, Hong Yang, Chinten J Lim, Gregor S Reid, Stuart E Turvey

**Affiliations:** 1 Department of Microbiology & Immunology, University of British Columbia, Vancouver, BC V6T 1Z4 Canada; 2 Experimental Medicine Program, University of British Columbia, BC Children’s Hospital, Vancouver, BC Canada; 3 Department of Pediatrics, BC Children’s Hospital, University of British Columbia, Vancouver, BC Canada; 4 Department of Respiratory Medicine, Shanghai First People’s Hospital, Shanghai Jiaotong University School of Medicine, Shanghai, China

## Abstract

Proteasome inhibitors have emerged as an effective therapy for the treatment of haematological malignancies; however, their efficacy can be limited by the development of tumour resistance mechanisms. Novel combination strategies including the addition of TLR adjuvants to increase cell death and augment immune responses may help enhance their effectiveness. Although generally thought to inhibit inflammatory responses and NF-κB activation, we found that under specific conditions proteasome inhibitors can promote inflammatory responses by mediating IL-1β maturation and secretion after TLR stimulation. This was dependent on the timing of proteasome inhibition relative to TLR stimulation where reversal of treatment order could alternatively increase or inhibit IL-1β secretion (*P* < 0.001). TLR stimulation combined with proteasome inhibition enhanced cell death in vitro and delayed tumour development in vivo in NOD SCID mice (*P* < 0.01). However, unlike IL-1β secretion, cell death occurred similarly regardless of treatment order and was only partially caspase dependent, possessing characteristics of both apoptosis and necrosis as indicated by activation of caspase-1, 3, 8 and RIP3 phosphorylation. Although stimulation of various TLRs was capable of driving IL-1β production, TLR4 stimulation was the most effective at increasing cell death in THP-1 and U937 cells. TLR4 stimulation and proteasome inhibition independently activated the RIP3 necroptotic pathway and ultimately reduced the effectiveness of caspase/necroptosis inhibitors in mitigating overall levels of cell death. This strategy of combining TLR stimulation with proteasome inhibition may improve the ability of proteasome inhibitors to generate immunogenic cell death and increase anti-tumour activity.

## Introduction

Since approval of the proteasome inhibitor bortezomib for clinical use in 2008, targeting of the ubiquitin–proteasome system has emerged as an effective strategy for treating haematological malignancies, including multiple myeloma and mantle cell lymphoma. However, the broader success of proteasome inhibitors has been limited by the development of resistance mechanisms as well as its relative inefficacy for other cancers such as solid tumours^1,2^ and acute myeloid leukaemia^3^. These limitations may be addressed by use of combination treatment strategies to increase response rates and efficacy of proteasome inhibitors.

Several studies have found that the ability of proteasome inhibitors to induce cell death can be augmented through co-stimulation of Toll-like receptors (TLRs). This includes TLR1/2 stimulation, which increases myeloma apoptosis at least partially through caspase-3 activation^4^, and increased cell death and anti-myeloma activity following TLR9 ligation in combination with bortezomib^5^. Although TLR monotherapy has long been suggested as a possible method to induce anti-tumour responses, TLR agonists have only seen limited clinical use in bladder and skin cancers (Bacille Calmette-Guérin and imiquimod, respectively)^6^. Instead, many studies have indicated that TLR agonists alone may have detrimental clinical effects by creating an inflammatory environment favourable for tumour growth, although whether the effects promote or inhibit tumour growth appears to depend on the specific TLR^7^. However, the potential capacity of TLR agonists to boost immune responses to tumour antigens continues to make them an attractive target in cancer therapy.

In addition to suppression of NF-κB activity^8–11^, proteasome inhibitors have been shown to impair cancer growth in a number of other ways. These include regulation of cell cycle progression, angiogenic factor expression and alterations in the balance of pro- and anti-apoptotic factors towards cell death^12^. Another promising characteristic of proteasome inhibitors is their ability to induce immunogenic cell death (ICD), which has been shown to elicit anti-tumour responses^13^. Bortezomib administration may improve natural killer cell recognition of tumour cells due to downregulation of cell surface HLA class I^14^, improve cytotoxic T cell function by upregulating Fas expression on solid tumours^15^ and increase the ability of dendritic cells to elicit T cell responses through cell surface expression of Hsp60 and Hsp90^16^. Conversely, other studies have found that proteasome inhibition may be immunosuppressive, reducing dendritic cell phagocytic capacity and maturation^17,18^. This has been supported by studies observing decreased production of IFNγ and IL-2 by T cells following bortezomib treatment^19^. Pairing TLR adjuvants with proteasome inhibition may therefore serve as a potential method to circumvent these immunosuppressive effects by eliciting additional stimulation of immune cells.

In this study, we investigated the effects of TLR adjuvant and bortezomib combination therapy on the production of IL-1β and cell death. We hypothesised that the addition of TLR adjuvants to proteasome inhibitors would augment ICD and stimulate immune responses through production of IL-1β. We provide evidence that co-treatment of myeloid tumour cells with TLR ligands in conjunction with proteasome inhibition can affect tumour growth by (1) modulation of IL-1β synthesis and maturation and (2) increasing caspase-dependent and caspase-independent modes of cell death.

## Results

### Proteasome inhibitors induce IL-1β secretion

**Fig. 1:**
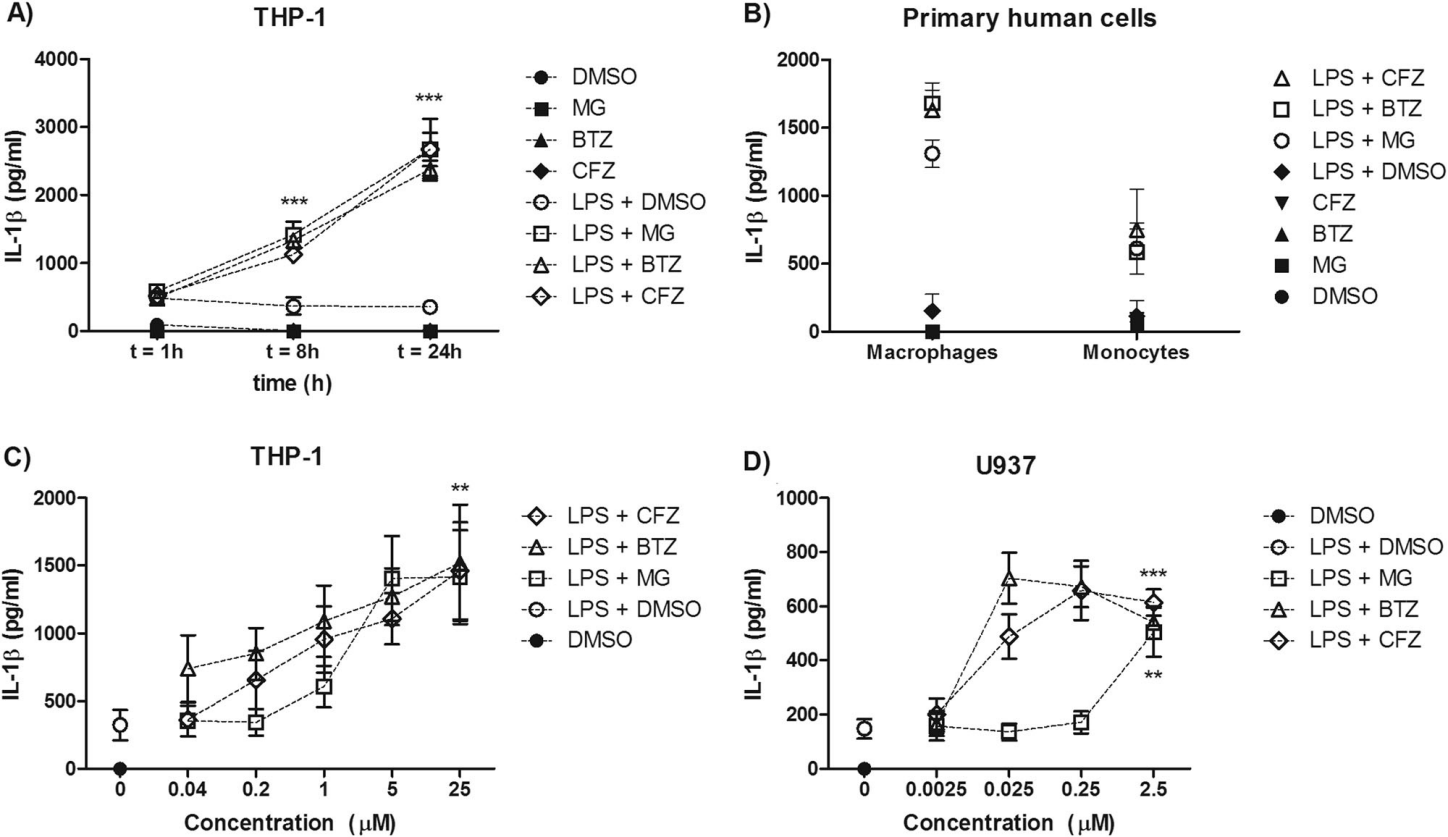
Proteasome inhibitors induce IL-1β secretion. **a** THP-1 cells were pre-treated with LPS (10 ng/ml) for 2 h prior to exposure to 5 μM of proteasome inhibitors (MG-132, bortezomib and carfilzomib). Supernatants were collected 1, 8 and 24 h after proteasome inhibition and assayed for IL-1β secretion. **b** Peripheral blood-derived human macrophages and monocytes were stimulated as in **a** and assayed for IL-1β secretion after 24 h. **c** THP-1 and **d** U937 cells were pre-treated with 10 ng/ml LPS for 2 h prior to proteasome inhibitor treatment. Supernatants were collected and assayed for IL-1β levels after 24 h. *n* = 3 for all experiments (independent experiments for cell lines, individuals for primary cells). Data were analysed using two-way ANOVA and the Bonferroni post-test. ** and *** indicate *P* < 0.01 and *P* < 0.001, respectively

Given the lack of clarity from the literature regarding potential pro- or anti-inflammatory activity of proteasome inhibitors, we first established whether or not proteasome inhibition in combination with a TLR agonist could consistently induce IL-1β production. To generate broadly generalisable results, we employed three different proteasome inhibitors (MG-132, bortezomib and carfilzomib) and we tested these inhibitors on both human primary blood cells (monocytes and M-CSF-derived macrophages) and two cell lines (THP-1 and U937) (Fig. 1a–d). After LPS priming, it was found that proteasome inhibition could induce significant IL-1β secretion in all the tested cell types by 24 h. The kinetics of IL-1β secretion were defined in THP-1 cells over 24 h (Fig. 1a). Enhanced IL-1β secretion was observed over various proteasome inhibitor concentrations, including those reported in the range of achievable plasma levels of bortezomib (MW = 384.2 g/mol) measured in patients: from 109 ng/ml (~283 nM) to 1300 ng/ml (~3.383 μM) at a treatment dose of 1.3 mg/m^2^ ^20^ (Fig. 1c, d). Concentrations of proteasome inhibitors used in this study were initially selected based on their ability to induce cell death in comparison to MG-132 (Supplementary Figure 1A) as the effect of inducing IL1β secretion was initially observed using this inhibitor. After 24 h of treatment, 5 μM of proteasome inhibitor was found to elicit an equivalent degree of cell death among all three inhibitors. Although 5 μM of bortezomib is a relatively high concentration for in vitro use, treatment at the lower concentration of 100 nM was subsequently found to similarly suppress proteasome activity in PBMC extracts (Supplementary Figure 1B).

### Proteasome inhibition induces processing of bioactive IL-1β

**Fig. 2:**
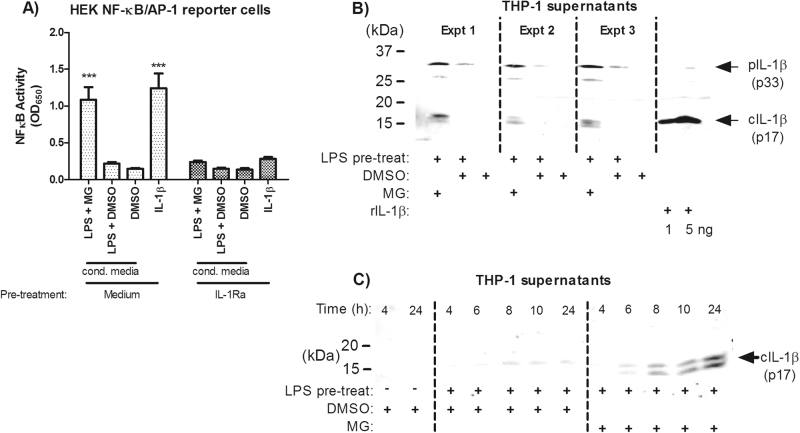
Proteasome inhibition induces processing of bioactive IL-1β. **a** Conditioned media from THP-1 cells pre-treated 2 h with LPS (10 ng/ml) and then stimulated with MG-132 (5 μM), LPS with vehicle or vehicle only for 24 h, were used to stimulate HEK293 null 1 (NF-κB/AP-1 reporter) cells (*n* = 3) in a 1:2 ratio with DMEM. As a control, 2 ng/ml of recombinant IL-1β was used to stimulate these cells in parallel. Responses were measured in the presence of either media or IL-1Ra (200 ng/ml) pre-treatment. **b** Supernatants from THP-1 cells stimulated as previously indicated for 24 h were blotted for IL-1β processing (*n* = 3). **c** THP-1 supernatants stimulated with LPS or LPS+MG were examined for the appearance of cleaved IL-1β over 24 h (*n* = 3). Data were analysed using two-way ANOVA and the Bonferroni post-test. *** indicates *P* < 0.001 compared to the IL-1Ra-treated condition

Proteasome inhibitors induce cell death which may result in pro-IL-1β release and quantification using ELISA may not necessarily differentiate between pro- and bioactive-cleaved forms of IL-1β. To address this possibility, we examined the capacity of the IL-1β secreted by THP-1 cells to stimulate an NF-κB response in cells expressing a reporter system for NF-κB/AP-1 activity. Conditioned media from THP-1 cells treated with LPS and MG-132 induced strong NF-κB/AP-1 activity which was abolished with IL-1 receptor antagonist (IL-1Ra) pre-treatment (Fig. 2a). Furthermore, examination of supernatants by immunoblot showed that while pro-IL-1β was also present in the supernatant, cleaved IL-1β (p17) was observed only in the LPS + MG-132 condition (Fig. 2b). Appearance of bioactive-cleaved IL-1β by immunoblot (Fig. 2c) corresponded with the temporal increases in secreted IL-1β observed by ELISA in Fig. 1a. Together, these data establish that combination therapy with a proteasome inhibitor and TLR agonist results in the secretion of bioactive IL-1β.

### Divergent effects of proteasome inhibition on IL-1β synthesis, degradation and secretion are dependent on relative treatment order

**Fig. 3:**
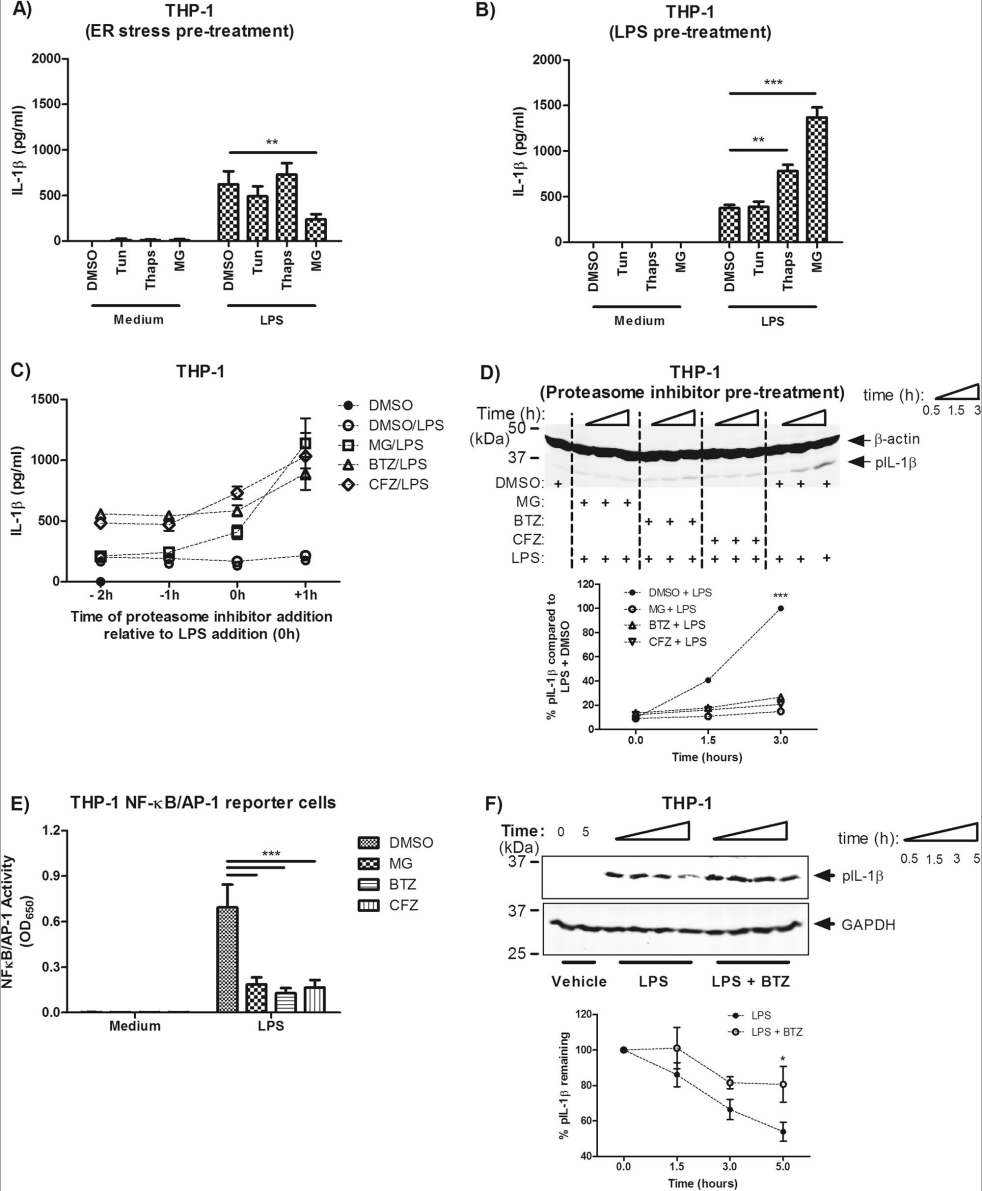
Divergent effects of proteasome inhibition on IL-1β synthesis, degradation and secretion are dependent on relative treatment order. THP-1 cells were exposed to the ER stressors tunicamycin (10 μg/ml), thapsigargin (10 μM) and MG-132 (5 μM) either 2 h **a** before or **b** after stimulation with LPS (10 ng/ml). **c** MG-132, bortezomib and carfilzomib (all 5 μM) were added to THP-1 cultures either before (2 h, 1 h), at the same time or 1 h after LPS stimulation (time = 0). Supernatants were harvested 24 h after stimulation. **d** Proteasome inhibitors (5 μM) were applied 2 h prior to LPS stimulation (10 ng/ml) and harvesting of cell lysates occuring at 0.5, 1.5 and 3 h post stimulation. Lysates were blotted for pro-IL-1β and quantified for its presence relative to LPS + DMSO. **e** THP-1 X Blue (NF-κB/AP-1 reporter) cells were treated as in **d**, but supernatants were collected after 6 h and assayed for SEAP activity overnight. **f** THP-1 cells were stimulated with LPS (1 ng/ml) for 4 h prior to removal of medium and treatment with bortezomib (5 μM) and/or cycloheximide (200 μg/ml). Lysates were collected at 0, 1.5, 3 and 5 h and blotted for pro-IL-1β to observe its relative degradation. Data were analysed using two-way ANOVA and the Bonferroni post-test. *, ** and *** indicate *P* < 0.05, *P* < 0.01 and *P* < 0.001, respectively. *n* = 3 for all experiments except **f**, where *n* = 4

Proteasome inhibitors are typically thought to mediate anti-inflammatory effects^12^ but they also activate the unfolded protein response^21^, which can have pro-inflammatory consequences. We compared the ability of MG-132 along with the ER stressors tunicamycin (10 μg/ml) and thapsigargin (1 μM) to induce IL-1β secretion by varying the timing of ER stressor treatment to occur either before or after LPS treatment. Interestingly, MG-132 treatment prior to LPS exposure inhibited IL-1β secretion (Fig. 3a), whereas treatment with LPS prior to MG-132 exposure resulted in an approximately threefold increase in secreted IL-1β over LPS alone (*P* < 0.001, Fig. 3b). This effect was also true for bortezomib and carfilzomib, where pre-treatment or even simultaneous treatment with LPS kept IL-1β production close to baseline levels but treatment after LPS priming increased IL-1β secretion (Fig. 3c). We subsequently found that proteasome inhibitor pre-treatment reduced pro-IL-1β synthesis (Fig. 3d) and that this seemed to correlate with decreased NF-κB/AP-1 activity (Fig. 3e). In contrast, proteasome inhibition also delayed degradation of existing cytosolic pro-IL-1β (Fig. 3f), potentially allowing for increased processing and maturation by caspases at later time points and may help to explain increased levels IL-1β secretion with LPS pre-treatment.

### IL-1β priming can occur through various TLRs, is limited by the presence of pro-IL-1β and correlates with cell death

**Fig. 4:**
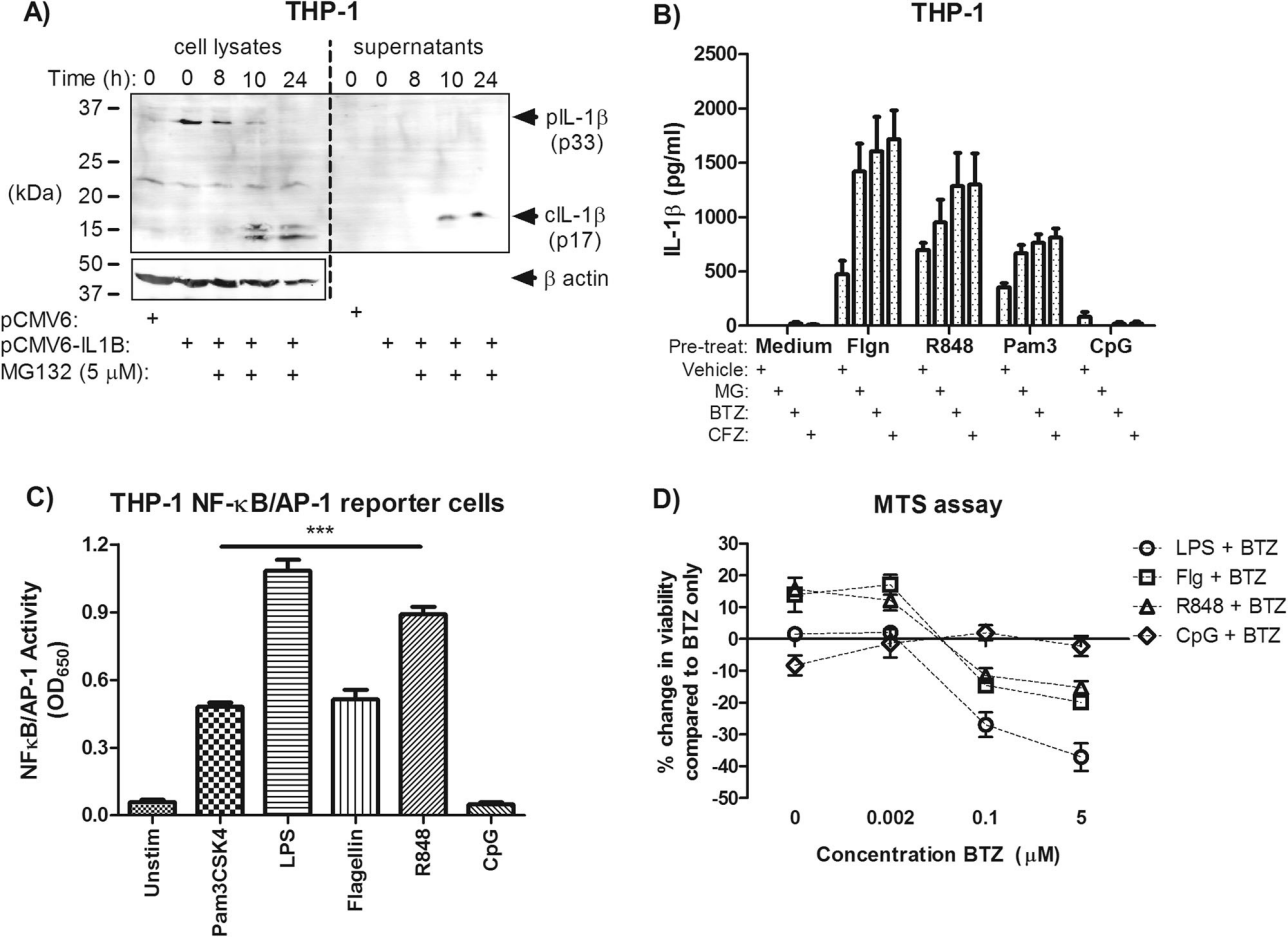
IL-1β secretion can occur with priming of various TLRs and correlates with cell death. **a** THP-1 cells transiently transfected with pro-IL-1β-expressing plasmid were treated with 5 μM MG-132. Lysates and supernatants were harvested at 0, 8, 10 and 24 h post exposure and blotted for IL-1β processing (data are representative of *n* = 3 experiments). **b** THP-1 cells were treated with 500 ng/ml flagellin, 100 ng/ml Pam3CSK4, 2.5 µg/ml R848 or 5 μM CpG DNA 2 h prior to proteasome inhibitor treatment for 24 h (*n* = 3). **c** THP-1 X-Blue (NF-κB/AP-1 reporter) cells were examined for the ability of TLR ligands to activate NF-κB/AP-1 activity over 24 h (*n* = 3–5). **d** LPS (10 ng/ml), flagellin, R848 and CpG-treated THP-1 cells (2 h) were exposed to 2 nM, 100 nM or 5 μM bortezomib and checked for viability after 24 h via MTS assay (*n* = 3). **d** Data were analysed using one-way or two-way ANOVA with Bonferroni post-test. *** indicates *P* < 0.05 and *P* < 0.001, respectively

The ability of proteasome inhibitors to directly induce pro-IL1β processing was established by transfecting THP-1 cells with a pro-IL-1β-encoding vector prior to proteasome inhibitor treatment (Fig. 4a). We further established that priming of IL-1β maturation can occur using a number of TLR agonists, including flagellin, R848 and Pam3CSK4 (Fig. 4b) as long as cells showed responsiveness to the TLR ligand in question (Fig. 4c). For example, THP-1 cells do not respond well to CpG stimulation and did not secrete IL-1β (Fig. 4b). This responsiveness also correlated with cell death, where flagellin and R848 increased cell death over bortezomib treatment alone quantified via 3-(4,5-dimethylthiazol-2-yl)-5-(3-carboxymethoxyphenyl)-2-(4-sulfophenyl)-2H-tetrazolium, inner salt (MTS) assay (Fig. 4d). Effects on cell death were further corroborated by staining THP-1 cells for annexin V/propidium iodide for analysis by flow cytometry. Combination treatment appeared to result in the largest accumulation of annexin V/propidium iodide double-positive cells over single treatments alone (Supplementary Figure 2A–C).

### Dissociation of IL-1β processing and cell death via caspase inhibition

**Fig. 5:**
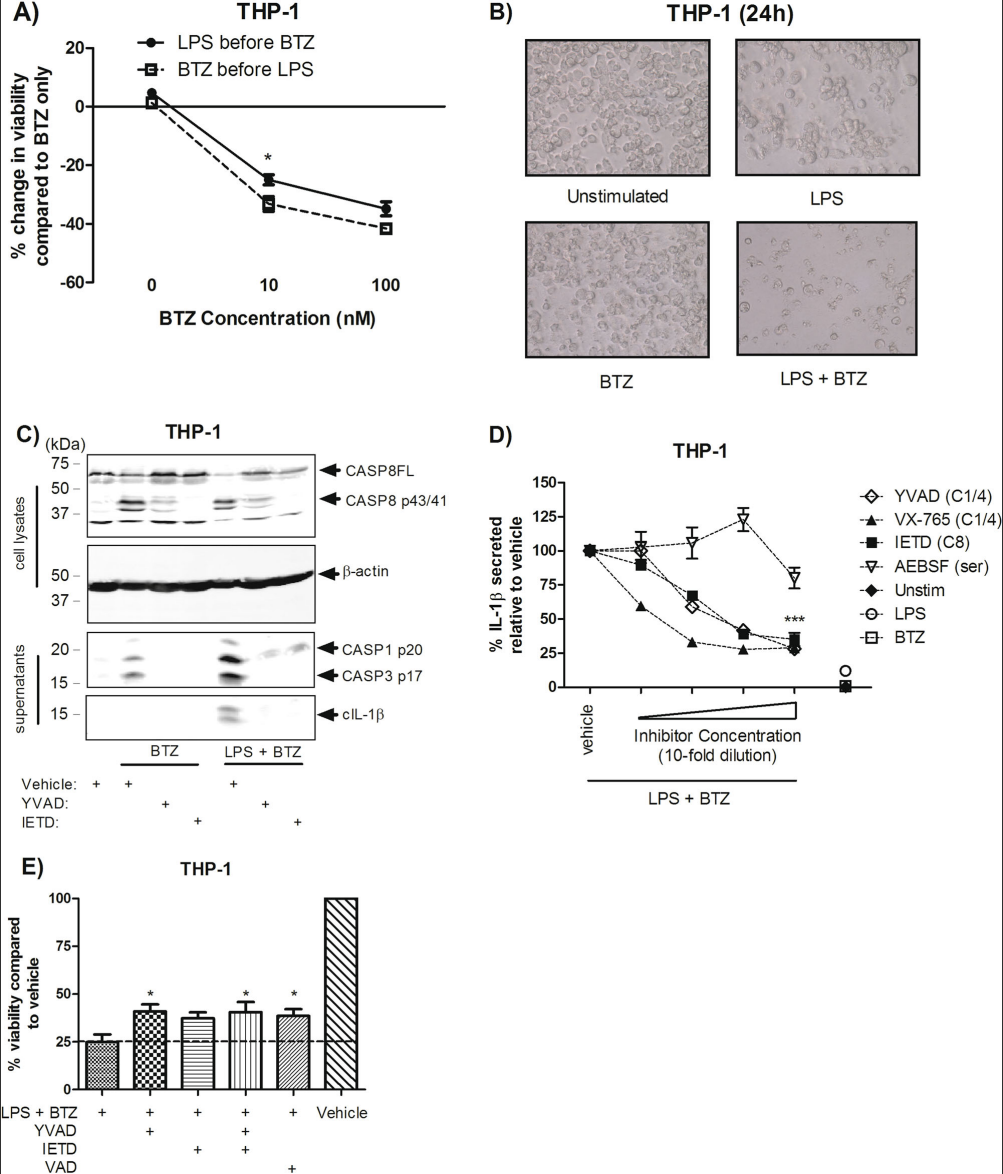
Dissociation of IL-1β processing and cell death via caspase inhibition. **a** THP-1 cells were stimulated with LPS either before or after bortezomib (10 nM, 100 nM) exposure and examined for cell viability after 24 h by MTS (*n* = 3). **b** Images of THP-1 cells after 24 h under each condition. **c** THP-1 cells treated with z-YVAD-fmk (20 μM) or z-IETD-fmk (20 μM) were blotted for the inhibition of caspase-8, caspase-1 and downstream caspase-3 and IL-1β processing in response to LPS (10 ng/ml) and bortezomib (5 μM) after 24 h (*n* = 3). **d** THP-1 cells were treated with various inhibitors in ten-fold dilutions, starting at 20 μM for z-YVAD-fmk, z-IETD-fmk, VX-765 and 300 μM for AEBSF (*n* = 3) prior to stimulation with LPS and bortezomib. Supernatants were examined for IL-1β. **e** Caspase inhibitors (20 μM) were examined for their ability to rescue cells from death after 24 h (*n* = 3) and were stimulated as in **c** and **d**. *, *** indicate *P* < 0.05, *P* < 0.001 by testing with two-way ANOVA and Bonferroni post-test

We next examined whether treatment order would affect cell death in a similar manner to IL-1β secretion. Unlike IL-1β secretion, cell death occurred with comparable kinetics regardless of treatment order, although cell death was slightly enhanced when cells were exposed to bortezomib (10 nM) before stimulation with LPS (Fig. 5a). Distinct morphological differences could be observed via brightfield microscopy with LPS + bortezomib treatment in comparison to either LPS or bortezomib alone (Fig. 5b). Cell death was characterised by the activation of caspases-1, 3 and 8 as measured in cell lysates and supernatants at 24 h (Fig. 5c) and activation of these caspases could be inhibited using the peptide inhibitors z-YVAD-fmk (caspase-1/4) and z-IETD-fmk (caspase-8). Curiously, although these inhibitors (along with VX-765, a caspase-1/4 inhibitor) inhibited IL-1β secretion in a dose-dependent manner (Fig. 5d), caspase inhibition was incapable of fully rescuing these cells from death as measured by MTS assay (Fig. 5e), indicating that distinct mechanisms regulate IL-1β maturation and cell death.

### The RIP3 necroptotic pathway is activated by combination therapy of bortezomib and LPS

**Fig. 6:**
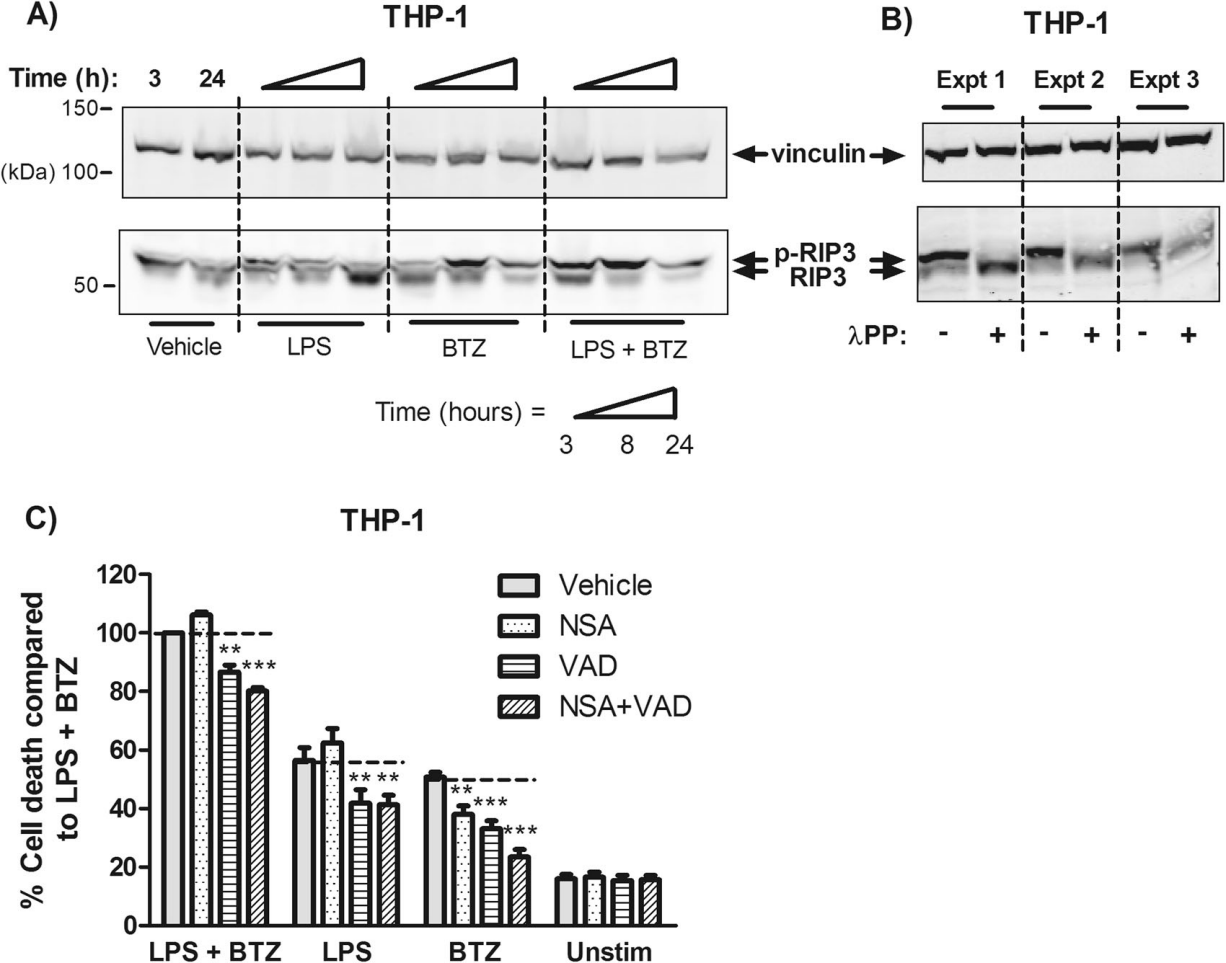
The RIP3 necroptotic pathway is individually activated by bortezomib and LPS. **a** THP-1 cells were treated with LPS (1 ng/ml) 2 h prior to treatment with bortezomib (100 nM), harvested at the indicated time points and blotted for RIP3 with vinculin as a loading control. **b** To show that the upper band observed for RIP3 corresponded with the phosphorylated form, lysates from THP-1 cells treated with LPS and bortezomib were treated with either protease/phosphatase inhibitor or lambda protein phosphotase (λPP). **c** THP-1 cells were treated with the necroptosis inhibitor, necrosulfonamide (4 μM NSA) or caspase inhibitor (20 μM z-VAD-fmk) under stimulatory conditions for 24 h. **, *** indicate *P* < 0.01, *P* < 0.001 by testing with two-way ANOVA and Bonferroni post-test

We subsequently investigated the role of necroptosis, a form of cell death known to involve inflammasome activation and IL-1β secretion^22^ which proceeds in a caspase-independent manner. In fact, Moriwaki et al. recently showed that proteasome inhibition could activate necroptosis through the RIP3 pathway in the absence of additional caspase inhibition^23^. We found that both TLR stimulation and proteasome inhibition could induce RIP3 phosphorylation, and increased overall RIP3 phosphorylation when combined (Fig. 6a). Because the antibody used for detection was not phospho-specific, we confirmed that the upper band did correspond to phorphorylated RIP3 using lambda phosphatase-mediated dephosphorylation (Fig. 6b). Quantification of necrosis/cell death by LDH assay showed that both caspase inhibition using z-VAD-fmk or necroptosis inhibition using necrosulfonamide (NSA) individually decreased bortezomib-mediated death (*P* < 0.01) and inhibition of both could account for the majority of bortezomib-mediated cell death. Although inhibition of both caspase activity and necroptosis improved suppression of cell death, this still only accounted for ~20% of cell death, suggesting the involvement of additional mechanisms.

### Combination therapy using a TLR4 agonist and bortezemib delays tumour growth in vivo

**Fig. 7:**
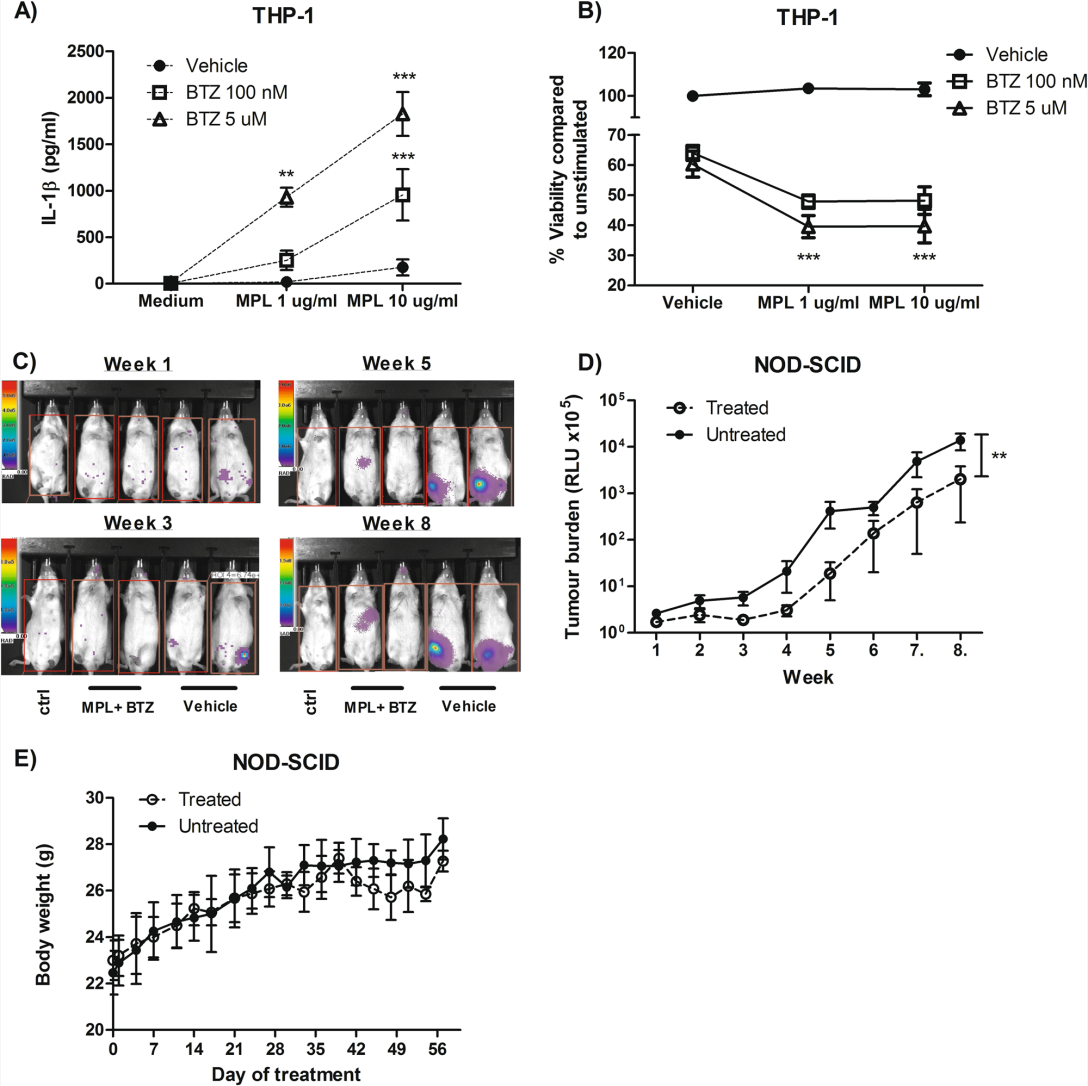
Combination therapy delays tumour growth in vivo. THP-1 cells stimulated with MPL and bortezomib were examined for **a** IL-1β secretion and **b** augmentation of cell death via MTS assay. Mice inoculated with THP-1 cells expressing GFP-luc were either treated with MPL + BTZ (*n* = 4) or vehicle (*n* = 3), imaged over 8 weeks for development of tumour signal **c**, and quantified **d** along with body weight **e**. **, *** represent *P* < 0.01, *P* < 0.001 using either a two-way ANOVA with Bonferroni post-test or the Wilcoxon signed rank test

Encouraged by the significant increase in tumour cell death we observed in vitro following combination therapy, we designed translational experiments to test this approach in vivo. We transduced THP-1 cells with a GFP-luciferase-expressing lentiviral vector, injected these cells into immunodeficient mice, and non-invasively quantified tumour development via repeated luminescent imaging over 8 weeks. To enhance translational potential of these experiments, instead of using LPS as the TLR agonist, we chose to use monophosphoryl lipid A (MPL) which is a less toxic derivative of LPS that is currently used as a vaccine adjuvant in humans^24^. Importantly, like LPS, we established that MPL combined with bortezomib induced both IL-1β secretion (Fig. 7a) and cell death (Fig. 7b). To determine if combination therapy was effective and tolerable, we treated mice with the combination of MPL and bortezomib mice over 8 weeks. Mimicking the effects of combined therapy that we found in vitro (Figs. 4 and 5), mice receiving combined therapy with MPL and bortezomib had lower tumour burden than untreated mice (*P* < 0.01) (Fig. 7c, d) and maintained similar body weight (Fig. 7e). In combination with our in vitro data, these in vivo results provide further evidence that combination therapy with proteasome inhibitors and TLR agonists enhances tumour cell death.

## Discussion

This study explored an immunomodulatory strategy to enhance the clinical efficacy of proteasome inhibitors such as bortezomib. Specifically, by using a therapeutic approach that combined a TLR agonist with a proteasome inhibitor, we found that (i) proteasome inhibitors regulate pro-IL-1β synthesis, degradation and maturation; (ii) unlike IL-1β maturation, cell death occurs in both caspase-dependent and independent manners and (iii) combination therapy inhibited tumour growth in vivo.

Although we found that primary cells (monocytes and MCSF-differentiated macrophages) secreted IL-1β after LPS priming and subsequent bortezomib treatment, it should be noted that we could not fully generalise our results for all cell lines tested. This was true for murine J774 and 289 cell lines, as well as the human HL-60 cell lines (Supplementary Figure 3A–C). In J774 cells, only poly(I:C) treatment led to increases in cell death as measured by supernatant LDH activity, whereas Pam_3_csk_4_ treatment actually seemed to inhibit it. Treatment of 289 cells with increasing levels of the TLR agonists MPL and Pam_3_csk_4_ led to a slight increase in cell death but only at high concentrations (μg/ml range) of agonist. Finally, HL-60 cells did not experience any significant augmentation in cell death with TLR pre-treatment in our hands. This may be due to a variety of reasons including the TLR expression profile on each of the tumour cell lines and their relative resistance to bortezomib. Depending on the mutational profile of these tumour lines, TLR signalling networks or mechanisms of cell death may also have been altered. Differences in the originating cell type may also play a role in whether or not IL-1β secretion would be observed, where cell lines originating from monocytes/macrophages may have a higher observable production of IL1β whereas tumour cells derived from lymphocytes may not intrinsically produce large amounts of IL-1β.

The role of TLR stimulation (and similarly, IL-1β, which shares the MyD88 signalling pathway) in cancer progression is somewhat contentious and appears to depend on the specific TLR in question. In general, TLR4 stimulation has been associated with worse disease, whereas TLR3 stimulation is typically associated with better outcomes^25^. This differential outcome is one of the reasons we selected MPL as the TLR agonist for our in vivo studies (Fig. 7), because even though MPL signals through TLR4, it has been suggested to bias towards TRIF-mediated, as opposed to MyD88-mediated signalling and so may have some of the benefits of TRIF-dependent tumour suppression^26^. TLR stimulation can result in NF-κB-mediated survival and the expression of pro-inflammatory mediators (including IL-1β and TNFα) which may aid in tumour cell adherence, angiogenesis and metastasis^7,27,28^. There is also evidence that stimulation through particular TLRs, such as TLR3 and TLR5, can induce anti-tumour responses and lead to increased tumour cell death^7^. TRIF, an adaptor protein recruited during TLR3 and TLR4 signalling, has been noted to interact with RIP1 and facilitate necroptosis through the recruitment of RIP3^29^. However, siRNA against TRIF was not sufficient to inhibit cell death in our model, while siRNA against MyD88 increased cell death (Supplementary Figure 4). Consequently, the successful use of TLR agonists to augment cell death will need to be tailored for the TLR response/expression pattern of the particular tumour cells.

Like TLR agonists, IL-1β possesses conflicting roles in cancer, where it is frequently associated with tumour progression^30,31^ but may also mediate anti-tumour responses through cytotoxic T cells^32^, enhancing effector functions and secondary responses^33^. IL-1R signalling also appears important for the function of the chemotherapeutic drugs doxorubicin and oxaliplatin in inducing IL-17A production by γδ T cells^17^, which may have anti-tumour functions. On the other hand, IL-1β may aid tumour spread by inducing angiogenesis^27^ and by promoting the development of myeloid-derived suppressor cells that can inhibit anti-tumour NK and cytotoxic T cell functions^34,35^. As the nature of our model only allowed us to examine the innate immune system, further studies are needed to evaluate the role of IL-1β on the adaptive immune response and tumour growth in the context of TLR + bortezomib combination treatment.

Proteasome inhibitors are widely used to suppress induced inflammatory responses by blocking the degradation of IκBα that is an essential step in the activation of NF-κB, but bortezomib has also been noted to increase basal NF-κB activity^36^. In fact, prolonged bortezomib treatment may increase basal inflammation which can eventually promote tumour spread. This was evident in a study showing that pro-inflammatory macrophages from bortezomib-treated mice could accelerate disease progression in a model of multiple myeloma, where tumour spread was increased by sustained proteasome inhibition^37^. Therefore, establishing an appropriate dose, duration of treatment cycle, as well as timing of TLR adjuvant administration will be critical in developing an optimal combined therapy using both a proteasome inhibitor and TLR agonist.

Informed by our data demonstrating that caspase inhibition could not completely inhibit cell death, we proceeded to examine the role of necroptosis—a form of caspase-independent cell death that is mediated by the RIP1 and RIP3 kinases^38^. Necroptosis is known to be tightly associated with caspase-8, inflammasome activation and IL-1β processing^39,40^, making it a good mechanistic candidate to explain our observations. It was recently reported that proteasome inhibition could induce necroptosis through the RIP3–MLKL pathway without additional caspase involvement^23^. LPS stimulation appeared to contribute to necroptosis in an additive manner, where LPS induced RIP3 phosphorylation independently of bortezomib. Inhibition of necroptosis using NSA in combination with caspase inhibition prevented the majority of cell death by bortezomib. However, the addition of LPS to bortezomib significantly enhanced cell death that could not be suppressed by NSA, even with caspase inhibition (Fig. 6c). We chose to chemically inhibit the MLKL pseudokinase as it serves to directly mediate necroptotic membrane disruption^41^, but because RIP3 itself may cause cell death and IL-1β maturation in the absence of MLKL^22^ other necroptosis components must also be tested.

In summary, our amalgamation of in vitro mechanistic studies with in vivo validation suggest that combination therapy with a proteasome inhibitor and TLR agonist has the potential to be a useful strategy for inducing tumour cell death in an inflammatory/immunogenic context.

## Materials and methods

### Cell culture and stimulations

Primary blood cells, THP-1 and U937 cells were cultured in RPMI1640 (Gibco) supplemented with 10% FCS, 2 mM sodium pyruvate and 1 mM l-glutamine. THP-1 cells were derived from a patient with acute monocytic leukaemia and U937 cells are myeloid cells derived from a patient with histiocytic lymphoma. Primary human macrophages were differentiated by magnetically separating CD14-positive cells (BD Biosciences) from peripheral blood mononuclear cells extracted on a Ficoll gradient (GE Healthcare) and subsequently differentiated over 10 days using 50 ng/ml M-CSF (Peprotech). They were stimulated the following day in the presence of 5 ng/ml M-CSF. Differentiation of both THP-1 and U937 cells were carried out using 50 ng/ml PMA for 24 h. Cells were rested for 36–48 h prior to stimulation. HEK293 null 1 (Invivogen) cells are HEK293 cells stably transfected with an NF-κB/AP-1 reporter. They were grown in high-glucose DMEM supplemented with 10% FCS, 2 mM sodium pyruvate and 1 mM l-glutamine, with the addition of 100 μg/ml normocin every third passage. MG-132 was from EMD Millipore, and bortezomib and carfilzomib were from Selleck chemicals. z-YVAD-fmk and z-IETD-fmk were from R&D systems. VX-765 was from Invivogen. AEBSF-HCl was from Enzo Lifesciences.

### NF-κB/AP-1 reporter assays

HEK293 null 1 cells were seeded at 7.5 × 10^4^ cells per well in a 96-well plate overnight. They were then treated with conditioned media from THP-1 cells that had been previously exposed to LPS and MG-132 either in the absence or presence of IL-1Ra pre-treatment. After 24 h, supernatants were collected and assayed for NF-κB/AP-1 activation by overnight incubation with Quanti-Blue substrate (Invivogen). THP-1 X-Blue (NF-κB/AP-1 reporter) cells are THP-1 cells that have been stably transfected with an NF-κB/AP-1 reporter. They were treated in the same was as THP-1 cells, with proteasome inhibition occurring 2 h prior to LPS treatment. After 6 h of LPS stimulation, supernatants were collected and incubated overnight with Quanti-Blue.

### Cycloheximide chase

After stimulating THP-1 cells with 1 ng/ml LPS for 4 h, media was replaced with either 200 μg/ml cycloheximide (EMD Millipore) or cycloheximide plus bortezomib (5 μM). Whole-cell lysates were collected at the indicated times.

### Immunoblotting

Cells were lysed in RIPA buffer (10 mM Tris, 150 mM NaCl, 1% Triton-X 100, 0.25% sodium deoxycholate, 0.1% SDS). Antibodies were from Cell Signaling Technologies (IL-1β, Caspase-1, β-actin, IκBα and RIP3) and R&D systems (Caspase-8 and vinculin). In total, 50–75 μg of protein was loaded per lane as determined by Bradford assay, depending on the abundance of the protein of interest. Blots were imaged on low autofluorescence PVDF (EMD Millipore) on a LICOR Odyssey scanner.

### RIP3 dephosphorylation

THP-1 cells stimulated with LPS + BTZ for 4 h were lysed in modified RIPA buffer containing 0.1% Triton-X 100 supplemented with proteasome inhibitor cocktail (Roche). In total, 70 μg protein was incubated with either HALT protease/phosphatase inhibitor cocktail (PIERCE) or 40 units of lambda protein phosphatase (NEB) for 30 min at 30 °C. Samples were run on a 10% polyacrylamide gel for prolonged separation.

### Conditioned media preparation

Conditioned media were prepared under the indicated conditions using 2 × 10^6^ cells/ml of media. After 24 h of stimulation, supernatants were collected and centrifuged at 2500 × *g* to remove non-adherent cells. Supernatants were stored in aliquots at −80 °C and were added at a final 1:2 conditioned media to fresh media ratio to HEK null 1 (NF-κB/AP-1 reporter) cells.

### ELISAs

ELISAs for IL-1β (eBioscience) were carried out according to the manufacturer’s instructions.

### Viability assays

3-(4,5-Dimethylthiazol-2-yl)-5-(3-carboxymethoxyphenyl)-2-(4-sulfophenyl)-2H-tetrazolium, inner salt (MTS) and lactate dehydrogenase (LDH) assays were from Promega and used according to the manufacturer’s instructions. Briefly, MTS substrate was diluted 1:9 in RPMI1640 with supplements and incubated with cells for 1–1.5 h at 37 °C prior to reading at 490 nm on a Spectramax PLUS384. LDH assays were performed using supernatants diluted in PBS and added to LDH substrate to achieve a final composition of ~15% supernatant. This was allowed to develop at room temperature for at least 20 min prior to reading at 490 nm.

### Cloning and transfections

THP-1 cDNA was used to PCR amplify *IL1B*. This was inserted into pCMV6-entry vector (Origene) using AsiSI and MluI restriction enzymes. The product was transfected into THP-1 cells (0.5 μg per 2 × 10^6^ cells) using solution SG and the Amaxa 4D nucleofector. Immediately after transfection, cells were differentiated overnight with 50 ng/ml PMA (~ 18 h). Cells were further rested 24 h prior to stimulation. Lentiviral vector expressing GFP and luciferase were transduced into THP-1 cells by overnight spinoculation at 1000 × *g*. Positive cells were positively selected by FACS (Aria II).

### NOD SCID mouse model

Non-obese diabetic/severe combined immunodeficient (NOD SCID) mice >5 weeks of age were injected with 5 × 10^5^ GFP-luc THP-1 cells via tail vein on day 0; 24 h later, they were treated with 0.75 mg/kg bortezomib and 0.67 mg/kg synthetic MPL (Invivogen) administered intra-peritoneally every 3 days over 8 weeks. Mice were imaged on an AmiX (Spectral Instruments Imaging) once per week with 15 mg/kg luciferin (GoldBio) administered intra-peritoneally.

### Ethics

PBMCs from healthy adult individuals were collected with informed consent under protocols approved by the UBC clinical research ethics board (H09-01192). NOD SCID mice were kept in accordance with the Canadian Committee on Animal Care and ethics were approved by UBC (protocol #A15-0187).

### Statistics

All experiments were performed at least three times unless otherwise indicated and analysed as stated in the figure legends with *, ** and *** indicating *P* values of *P* < 0.05, *P* < 0.01 and *P* < 0.001, respectively. Error bars indicate standard error of the mean, and statistical comparisons were typically made only to the control group unless stated otherwise.

## Electronic supplementary material

Supplemental figures and figure legends
