# Supplementary material for: Combination therapy with proteasome inhibitors and TLR agonists enhances tumour cell death and IL-1β production
Source: Cell Death Dis. 2018 Feb 7;9(2):162. doi: 10.1038/s41419-017-0194-1 (PMC5833743; doi:10.1038/s41419-017-0194-1)
Supplement: Supplementary file 1 — Supplemental figures and figure legends [file 41419_2017_194_MOESM1_ESM.docx]

**SUPPLEMENTARY FIGURES**

**Combination therapy with proteasome inhibitors and TLR agonists enhances tumour cell death and IL-1β production.**

Anthony C Tang^1^, Seyed M Rahavi^2^, Shan-Yu Fung^3^, Henry Y Lu^2,3^, Hong Yang^4^, Chinten J Lim^2,3^, Gregor S Reid^2,3^,andStuart E Turvey^1,2,3^

^1^Department of Microbiology & Immunology, University of British Columbia, British Columbia, Canada

^2^Experimental Medicine Program, University of British Columbia and BC Children’s Hospital, Vancouver, British Columbia, Canada

^3^Department of Pediatrics, BC Children’s Hospital, University of British Columbia, Vancouver, BC, Canada

^4^Department of Respiratory Medicine, Shanghai First People's Hospital, Shanghai Jiaotong University School of Medicine

**Key Words:** Proteasome inhibitors, IL-1β, Inflammation

**Corresponding Author:** Stuart Turvey MB BS DPhil FRCPC

BC Children’s Hospital

950 West 28 Avenue

Vancouver BC V5Z 4H4 CANADA

Ph: 604 875 2345 x5094

Email: [sturvey@cw.bc.ca](mailto:sturvey@cw.bc.ca)


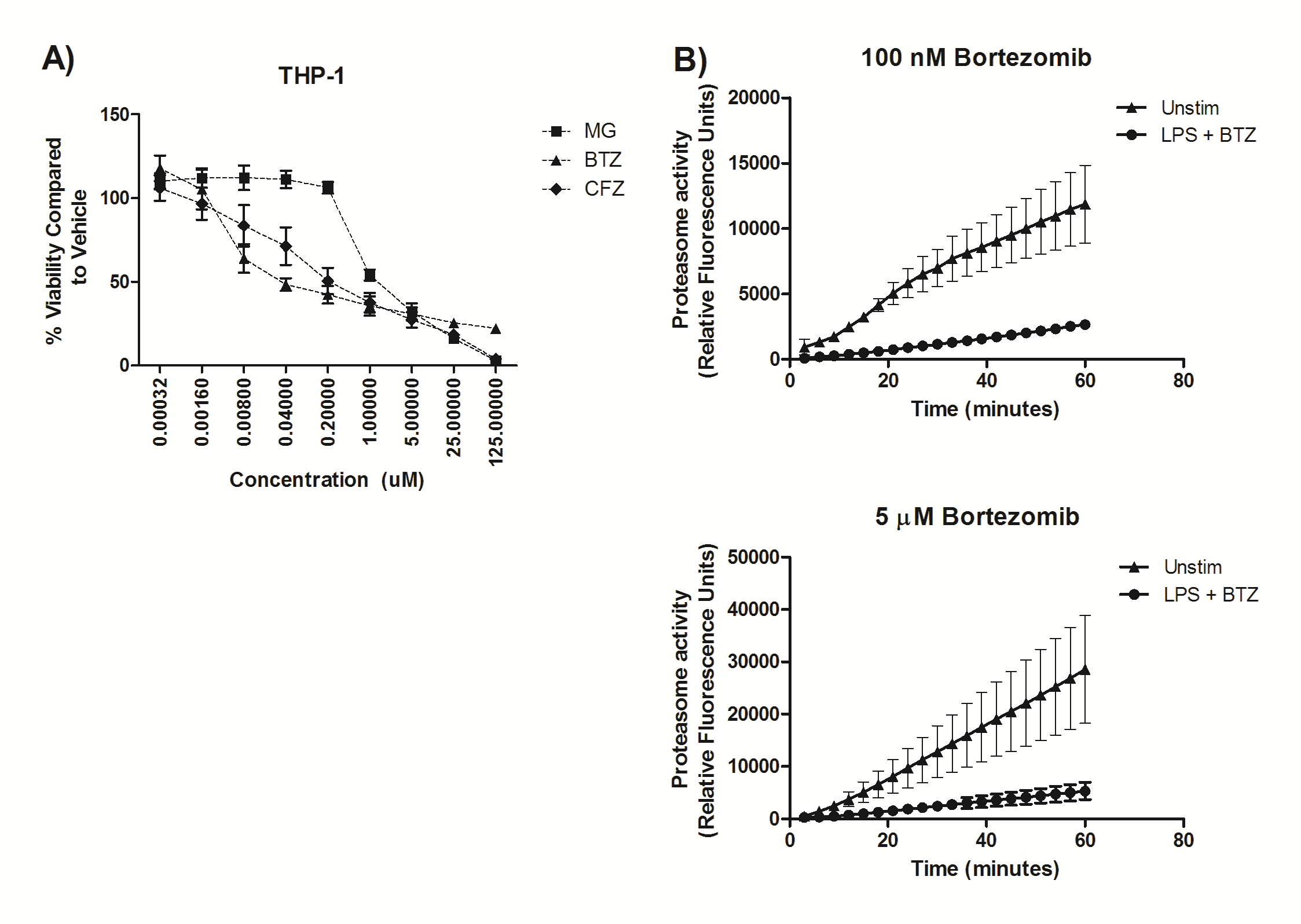


**Supplementary Figure 1: Dose response of proteasome inhibitors on cell death and proteasome function**

(A) THP-1 cells were treated with either bortezomib, carfilzomib, or MG-132 in a 5-fold dilution series starting at 125 μM. After 24 hours, cells were examined for overall cell death using the MTS assay. (B) PBMCs were treated with LPS (1 ng/ml) and bortezomib (100 nM or 5 μM) for 3 to 4 hours prior to collection and lysis in 0.5% Triton-X.10 μg of lysate was incubated with50μM of a fluorescent peptide (suc-LLVY-amc) substrate (ENZO lifesciences) and compared with untreated lysates.

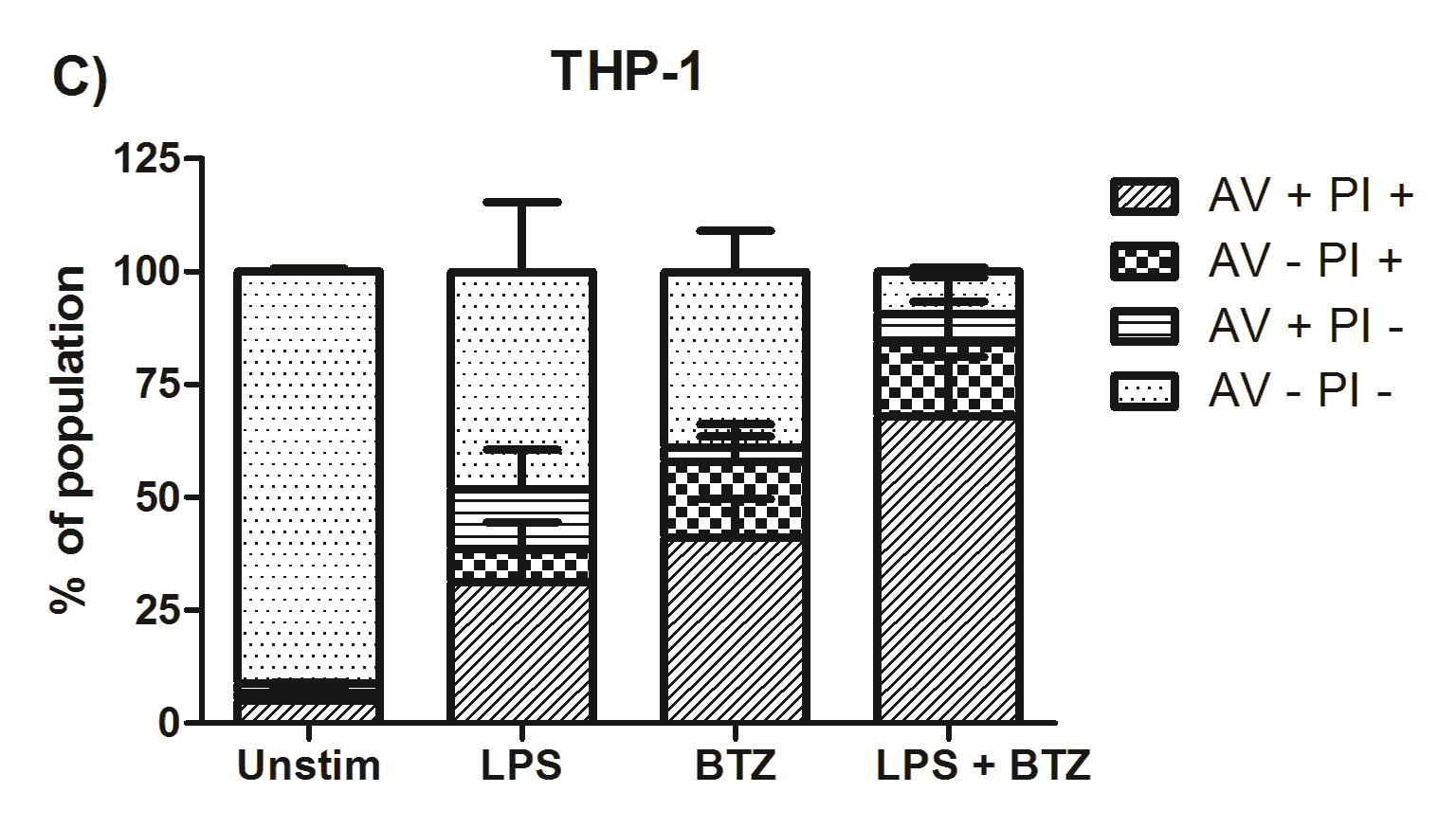


**Supplementary Figure 2: Combination treatment increases apoptosis and necrosis markers in THP-1 cells**

THP-1 cells were treated for 24 hours under the conditions listed, after which they were collected and analyzed by flow cytometry on an LSRII for concurrent annexin V and propidium iodide staining. A) and B) show data from a representative experiment while C) shows the mean ± SEM % of cells staining positive for both annexin V and propidium iodide, positive for only one cell death marker, or negative for both (n = 3).


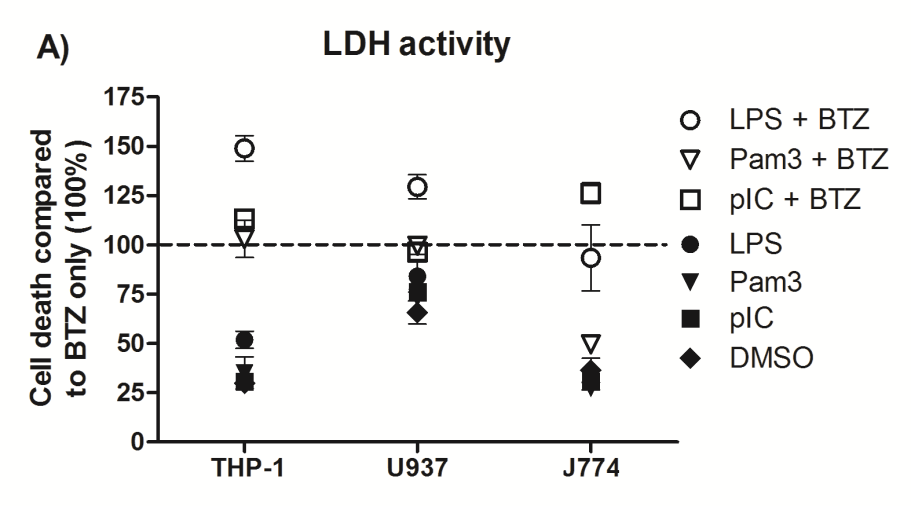


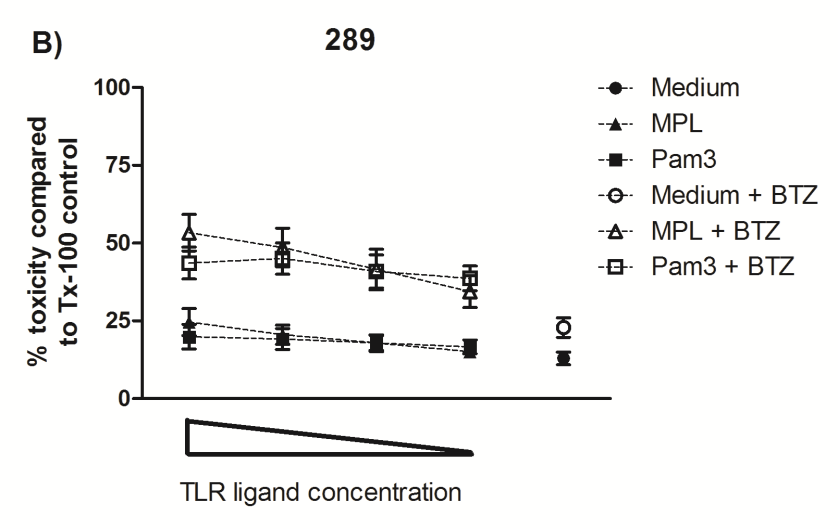


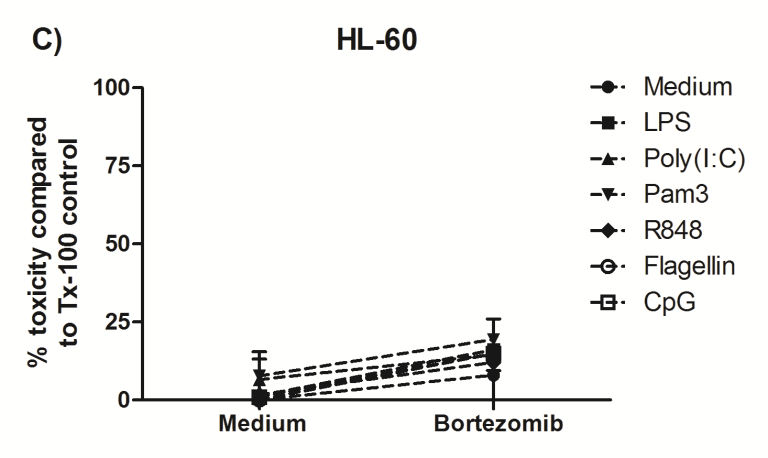


**Supplementary Figure 3:**

(A) J774 murine macrophages (n=3) were cultured in RPMI1640 supplemented with 10% FCS and stimulated as for THP-1 cells using the indicated TLR agonists prior to treatment with bortezomib. (B) 289 cells (n = 3) were cultured in RPMI1640 with 20% FCS and 100 μM 2-mercaptoethanol and subsequently pre-treated with TLR agonists (2-fold dilution series starting at 10 μg/ml MPL or 15 μg/ml Pam_3_csk_4_) in combination with 10 nM bortezomib over 24 hours. (C) HL-60 cells, derived from a patient with acute promyelocytic leukemia, did not display increased cell death in response to a number of TLR agonists combined with bortezomib (10 μM) (n = 2). We did not measure significant IL1β secretion from any of these cell lines (not shown).

**
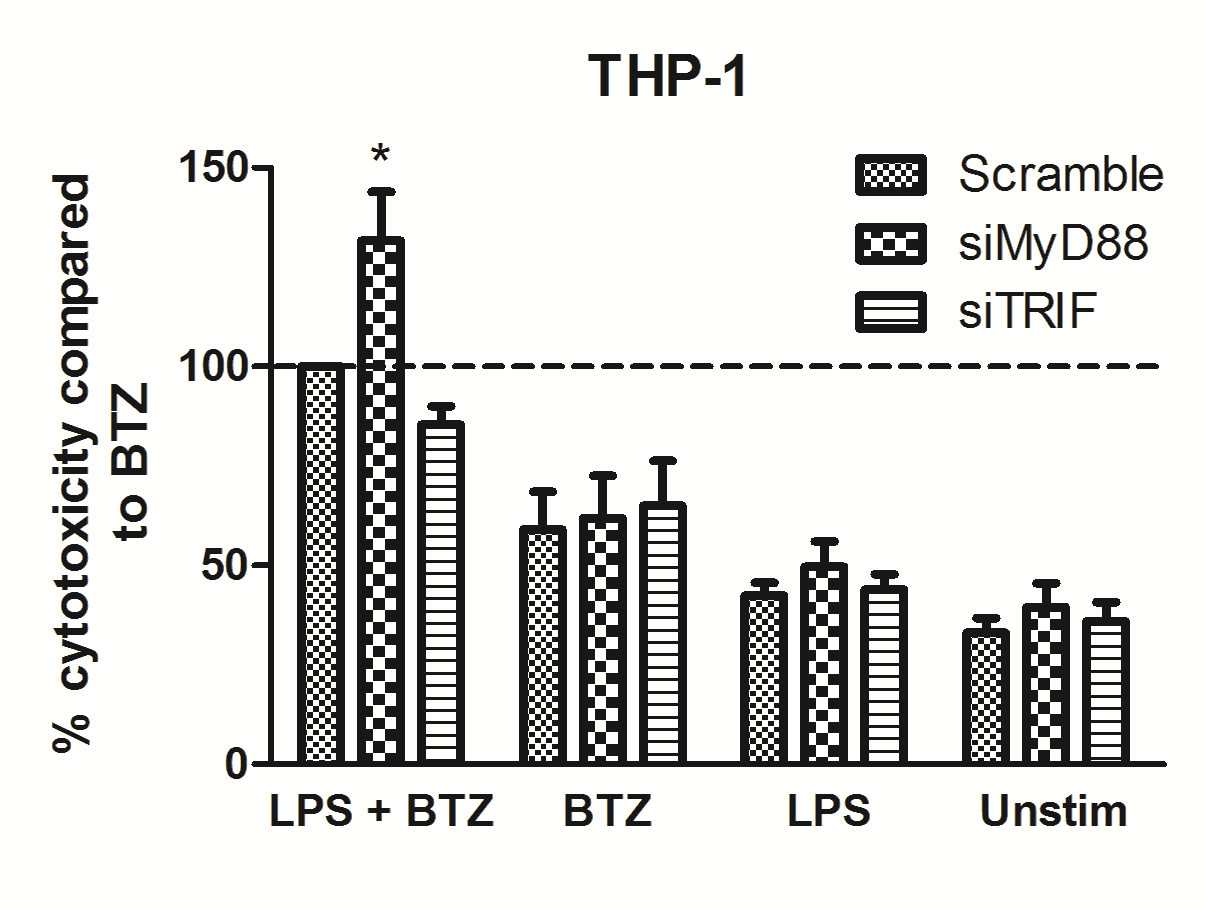
**

**Supplementary Figure 4:**

THP-1 cells were transfected (4D nucleofector, SG solution) with 500 nM of ON-TARGETplus siRNA against MyD88 and TRIF (Dharmacon) were transfected per 2 x 10^6^ cells in 100 μl of SG solution. Cells were differentiated using PMA and rested for 48 hours prior to stimulation with the conditions above (1 ng/ml LPS, 100 nM bortezomib). After 24 hours, supernatants were collected and analyzed via LDH assay.
